# Returning to work with long covid in the UK during lockdown and other COVID-19 restrictions: A qualitative study

**DOI:** 10.1371/journal.pone.0307062

**Published:** 2024-08-12

**Authors:** Clement Boutry, Priya Patel, Jain Holmes, Kathryn Radford, Charlotte E. Bolton, Nikos Evangelou, Roshan das Nair, Richard Morriss

**Affiliations:** 1 School of Medicine, University of Nottingham, Nottingham, Nottinghamshire, United Kingdom; 2 National Institute for Health and Care Research, Applied Research Collaboration East Midland, University of Nottingham, Nottinghamshire, United Kingdom; 3 NIHR Nottingham Biomedical Research Centre, Nottingham, United Kingdom; 4 Centre for Respiratory Research, Translational Medical Sciences, School of Medicine, University of Nottingham, Nottingham, United Kingdom; 5 Health Division, SINTEF, Trondheim, Norway; 6 Nottinghamshire Healthcare NHS Foundation Trust, Nottingham, United Kingdom; University of Toronto, CANADA

## Abstract

Few previous studies have considered the experiences of people with long covid returning to work beyond symptoms in terms of employer and other support factors. The aim of this research was to understand the experience of returning to work for those with long covid symptoms in contrast to the non-long covid group who had not experienced COVID-19 during the time restrictions were imposed to limit the spread of COVID-19 infection. Twenty-one participants with long covid and 13 participants who had not had COVID-19 were interviewed. Themes were identified from transcripts using framework analysis. Participants with long covid experienced difficulties returning to work, particularly from fatigue, cognitive symptoms and breathlessness. Symptoms were heterogeneous and fluctuated in severity over time. A lack of understanding from colleagues and managers sometimes led to a premature return to work without adequate support, associated with further long covid relapse. Outside-of-work, support was salient for a successful return to work. The standard phased return offered by employers may be too short and rigid for some people with long covid. They may benefit from a tailored intervention to support a return to work that targets symptom management, and engages with work colleagues, managers, and family members.

## Introduction

By March 23^rd^, 2023 the World Health organization (WHO) had reported 761,071,826 global cumulative cases of COVID-19 [1]. COVID-19 is caused by a Severe Acute Syndromes (SARS)-CoV2 acute infection. It can cause multiple symptoms, including breathlessness, fever, cough, and loss of sense of smell and taste [2,3] of varying severity depending on virus strain and individuals’ vaccination status, and has led to huge numbers of people being hospitalised, sometimes requiring ventilatory support, and in worst cases leading to death.

For survivors of acute infection, persisting symptoms have led to considerable morbidity. The National Institute for Health and Care Excellence (NICE) defines the commonly used term “long covid” as when signs and symptoms continue or develop after acute COVID-19 infection, thereby including both ongoing symptomatic and post-COVID-19 syndrome [4]. More women than men experience long covid, with increased risk with age [5–7]. Other characteristics such as obesity, smoking, and hospitalisation, in addition to being of white ethnicity also affect the risk of persistent symptoms [8]. A review categorised the range of symptoms as cardiopulmonary, naso-oropharyngeal, musculoskeletal and neuropsychological, in addition to other multiple symptoms such as dizziness and headaches [9].

A meta-analysis [10] estimates that 80% of patients who had COVID-19 still experience symptoms two weeks after infection, whilst recovery and symptom intensity vary greatly from one patient to another. As of March 5th, 2023, around 1.9 million individuals in the United Kingdom (UK) are estimated to be facing symptoms associated with a previous COVID-19 infection—within this group, 69% have been enduring these symptoms for at least one year [11]. Although physical and mental fatigue are common consequences of viruses and other inflammatory disorders (e.g., multiple sclerosis), there are many unusual neuropsychiatric features of COVID-19 including depression, auditory hallucinations, health anxiety with obsessive-compulsive features, delirium and isolated neurological symptoms thought to be due to micro clots, such as difficulty with speech, coordination or movement [12,13]. Some have now had these problems for over two years without improvement, with symptoms substantially interfering with everyday life, such as vocational and occupational activities [14]. Functional deterioration following critical illness may take months or years to resolve [15] with some patients not returning to their pre-illness status [16], which is evidenced by previous research focusing on SARS outbreaks with chronic fatigue residual symptoms lasting even four years later [17]. In fact, the COVID-19 pandemic has had a considerable impact on work and further widened the employment gap between disabled and non-disabled people [18]. Since the pandemic, the number of long-term sicknesses has greatly increased [19]. One national survey in 2021 indicated 600,000 people stopped working or reduced their hours due to long covid or the fear of the virus [20].

Returning to work after a critical illness is often associated with recovery [16] and improves physical/psychological health, quality of life, and financial stability [16,21]. However, critical illness survivors face increased risk of job loss and worsened employment status [22]. Unfortunately, post-viral syndromes are not well understood [23,24], and workers are often pressured to return to work (RTW) because of moral judgements and a lack of understanding or acknowledgement of the person’s symptoms [25]. In previous coronavirus outbreaks, only 78% of SARS survivors had returned to work within 24 months of infection [26], which is in line with what has been witnessed with COVID-19 [27–29], including those who had experienced mild symptoms during infection [30,31]. Contributing elements to the struggle in returning to work include fatigue, poor mental functioning via reduced attention/concentration and speed of processing, breathlessness, and anxiety caused by the traumatic memories of the coronavirus infection [32]. However, the interaction and impact of these elements in the context of COVID-19 are not yet fully understood.

Recent research investigated barriers to returning to work after COVID-19 and found that fatigue, physical symptoms, and difficulties with concentration [33] were the main obstacles to RTW. However, these were complicated by restrictions applied to whole population to combat the spread of COVID-19 infection. To combat the COVID-19 pandemic, the UK and other countries had restrictions in place, such as travel bans, social limitations, social distancing, and requirements to wear face masks. Through isolation, working from home (WFH), home schooling and job-related pressures (such as increase in workload), furlough schemes (whereby employees cannot continue to work for their employer but retain employment status and rights) and risk of job loss [33], there were considerable changes to work and personal life even for those who did not get COVID-19 [34]. It is unclear to what extent the pandemic and the associated public health restrictions are responsible for some of the work difficulties experienced by those with long covid. Furthermore, factors that might facilitate return to work under these restrictions also require exploration.

Therefore, we interviewed people with long covid and those who did not contract COVID-19, who were in work or seeking to return to work during the period of these COVID-19 restrictions in the UK. This comparison highlights important shared experiences due to the public health restrictions but reveals the unique barriers and enablers for RTW for those with long covid, which continue long after the pandemic.

### Aims

1. To understand the experiences of people with long covid in relation to work and return to work.
2. To ascertain the physical, mental, and vocational factors that are barriers or enablers to returning to their normal occupational or other vocational activities.

## Method

### Study design

A framework qualitative analysis of single semi-structured interviews with people with long covid covering their experiences of their work and return to work during public health restrictions to restrict COVID-19 infection, to identify barriers and facilitators to their return to work, and from people with no experience of COVID-19 infection (non-long covid group), to provide contextual information on the conditions of work at the time of restrictions due to COVID-19.

Ethical approval was provided by the University of Nottingham REC committee (ref: REC ref no: 143–1220). Written, informed consent was received from all the participants prior to starting interviews.

### Participants

We recruited participants who were aged between 18–65 years, able to give informed consent and to speak, read and write in the English language. In addition, those with long covid had to have previously tested positive for COVID-19, and been (self)employed, in education or voluntary work at the time of infection, and not returned to work/education at least a month after infection.

The non-long covid group participants were (self)employed, in education or voluntary work as of 01/03/2020, deemed the start of the COVID-19 pandemic in the UK [35]. They had had no active physical or mental health problems for two years and no suspected previous COVID-19 infection symptomatically or through a positive COVID-19 test.

### Recruitment strategy and procedure

We recruited participants between 21/03/2021 and 28/10/2021. We used convenience sampling to recruit participants through a survey assessing participants eligibility to take part in the study. A link to the survey was shared on social media, including Twitter^®^ and LinkedIn^®^ using hashtags or tagging tools relevant to the study (e.g., #longcovid, #returntowork), and on appropriate Facebook^®^ groups (e.g., long covid support group). In addition, National Health Service (NHS) clinics and services relevant to COVID-19 were contacted and encouraged to share the survey link with their patients and networks. The survey link was emailed to clinics and health centres supporting the project so that they might share with their network and patients. In addition, the research team shared the link within their own networks.

The survey was developed using REDCap (www.project-redcap.org). Participants accessing the survey were introduced to the study and its purpose before providing informed e-consent. Once eligibility was confirmed, participants provided demographic information and contact details for the interview. On average, the whole process from reading the information sheet to providing their contact details took 10 minutes.

### Interviews

The interviews were arranged within seven days of obtaining consent to participate and were conducted by a researcher over the phone or Microsoft Teams, and recorded on a password protected Olympus DS-7000 digital recorder. The interviewer was a male research assistant employed by the University of Nottingham, had an MSc in psychology and experience in conducting qualitative interviews in the context of research. Interviews were semi-structured and guided by an interview schedule. The interview schedule was developed based on existing research on long covid, experiences of returning to work after critical illness, and the aims of the study. During the interviews, participants in the long covid group were asked about their difficulties returning to work, the support they received, and were requested to reflect on what was helpful/unhelpful. The non-long covid group was asked similar questions but in relation to the pandemic and confinement/social isolation/quarantine (see Supplementary information 1 in S1 File). Interviews were transcribed using a professional transcriber. Upon review of the anonymised transcripts, within seven days of the interview, the original recordings were erased. Throughout the length of the study, only the interviewer had access to information that could identify participants.

### Data analysis

Interview data were analysed using framework analysis whereby data is summarised into a matrix to analyse data by case and code in a systematic way [36]. This method allowed us to integrate existing relevant frameworks, which can be refined during the analysis stage. Our original framework was created based on the brief International Classification of Functioning, Disability and Health (ICF) core set for vocational rehabilitation encompassing three overarching themes (activities & participation, environmental factors, body functions; World Health Organisation, 2007). Data were entered onto Nvivo v12 (www.qsrinternational.com) for analysis. Following data familiarisation, codes identified from line-by-line coding were mapped onto the brief ICF categories by one researcher (CB). During the coding stage, quotes that did not fit within the Brief ICF three categories (i.e., activities and participation, body functions, and environmental factors) taken from the comprehensive ICF core set were added to the brief ICF core set: (1) Economic self-sufficiency d890, (2) Product and technology for employment e135, and (3) Emotional functions b152. Data saturation was deemed reached as no new or meaningful codes emerged from the analysis. Upon coding completion, a matrix was generated based on the coding of the brief ICF core set such that items were grouped into meaningful themes and subthemes based on their content. The matrix was reviewed by two researchers (CB and PP) to ensure consistency in the overall coding and presented to the research team and a panel of people with lived experience of long covid, and the content discussed to refine the themes and subthemes and their content.

## Results

In total, 34 participants were interviewed. Interviews lasted between 8.35min and 26.10min. Twenty-one interviews were from the long covid group and 13 from the non-long covid group.

The mean age of respondents was 40.1 years (SD = 9.0), 85.3% (n = 29) were women, and 82.4% (n = 28) were white (British or other). Overall, 97.1% participants (n = 33) were in employment or self-employed, and 2.9% (n = 1) were students. The long covid and healthy non-long covid groups were matched for age, gender, and occupation (refer to Tables 1 and 2).

**Table 1:** Participant demographics.

Participant	Group	Age	Gender	Job	Ethnicity
1	Long covid	52	Woman	Healthcare professional	White British
2	Long covid	43	Woman	Associated healthcare professions	White British
3	Long covid	44	Woman	Veterinary surgeon	White Other
4	Long covid	37	Woman	NK	White British
5	Long covid	49	Woman	Associated healthcare professions	White British
6	Long covid	36	Woman	School worker	White British
7	Long covid	40	Man	Healthcare professional	White Other
8	Long covid	31	Woman	Healthcare professional	White British
9	Long covid	28	Woman	Healthcare professional	White British
10	Long covid	41	Woman	NK	White British
11	Long covid	19	Woman	Student	Other ethnic group
12	Long covid	39	Woman	Healthcare professional	White British
13	Long covid	32	Woman	Healthcare professional	White British
14	Long covid	29	Woman	Healthcare professional	White British
15	Long covid	41	Woman	Healthcare professional	White British
16	Long covid	52	Woman	Associated healthcare professions	White British
17	Long covid	26	Man	Corporate pension actuary	White British
18	Non-long covid	39	Man	IT manager	White British
19	Long covid	58	Woman	School worker	White British
20	Long covid	43	Woman	School worker	White British
21	Long covid	55	Woman	Healthcare professional	White British
22	Long covid	44	Woman	Associated healthcare professions	White British
23	Non-long covid	45	Woman	Healthcare professional	White British
24	Non-long covid	38	Woman	School worker	African/Caribbean/Black/ Black British
25	Non-long covid	39	Woman	Healthcare professional	White British
26	Non-long covid	29	Woman	Associated healthcare professions	Mixed/multiple ethnic group
27	Non-long covid	50	Woman	Healthcare professional	Mixed/multiple ethnic group
28	Non-long covid	40	Woman	School worker	African/Caribbean/Black/ Black British
29	Non-long covid	28	Woman	Healthcare professional	Asian/Asian British
30	Non-long covid	35	Woman	School worker	White British
31	Non-long covid	42	Woman	School worker	White British
32	Non-long covid	54	Woman	School worker	White British
33	Non-long covid	42	Man	Builder	White British
34	Non-long covid	42	Man	Technical sales manager	White British

NK = Not Known.

**Table 2:** Demographics within groups.

Group	Average age (standard deviation)	% women	% in office-based roles	% in Healthcare roles	% in teaching roles	% returning to standard environment after lockdown
Long covid	40 (10.1)	90.5	23.8	42.8	14.3	76.2
Non-Long covid	40 (7.2)	76.9	30.8	30.8	23.1	76.9

**Fig 1:**
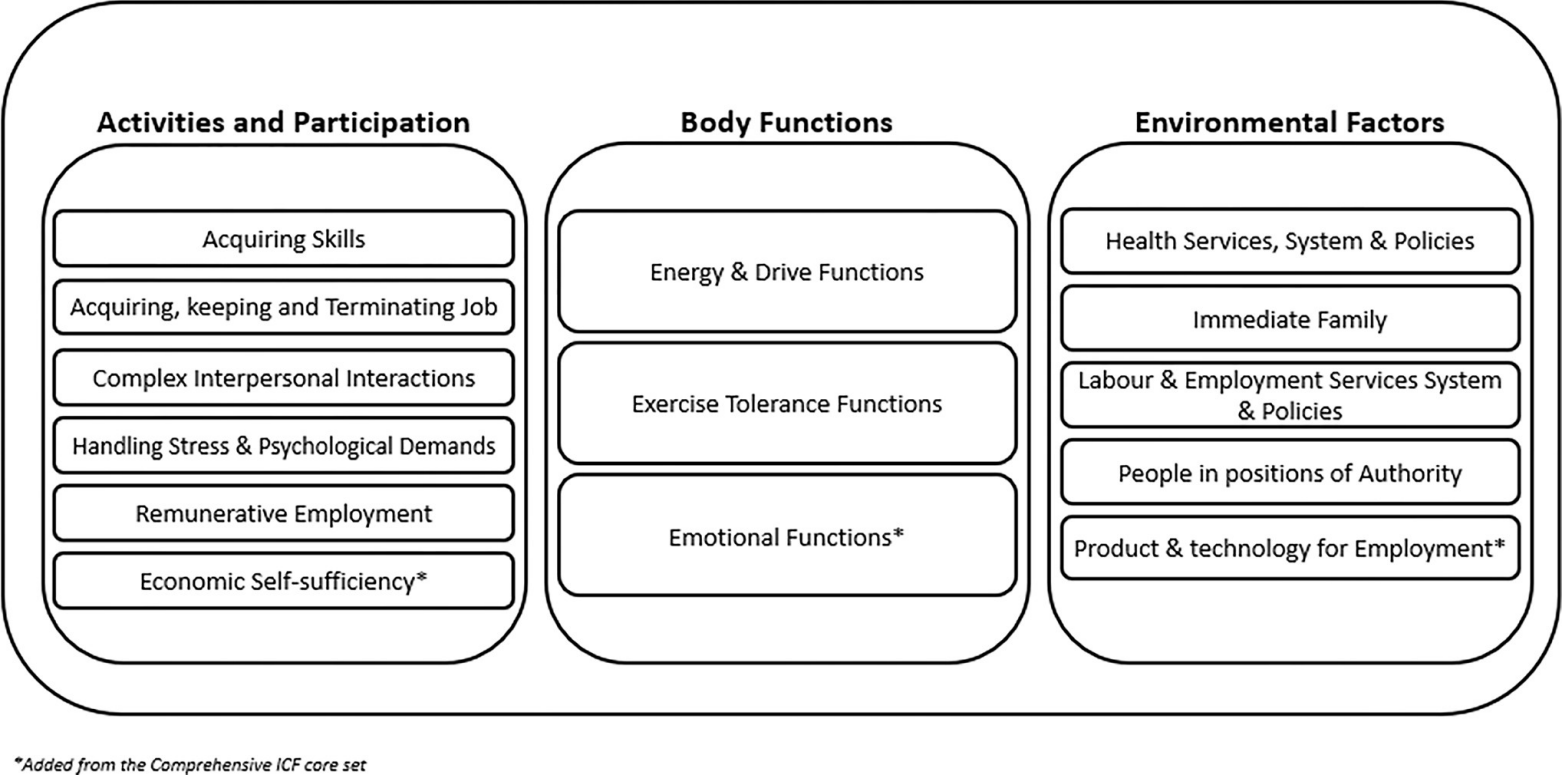
Brief ICF core set and added ICF categories used during analysis.

Within the ICF core set, three main themes and subthemes were reported to describe the experience of RTW following long covid or as a non-long covid participant during the pandemic. The Brief ICF core set’s three primary themes and their respective subthemes, along with the inclusion of three additional ICF subthemes (namely, emotional functions and Product & Technology for Employment) due to their relevance to the transcript content, is presented in Fig 1. Links between the ICF core set and the themes and subthemes can be found in Supplementary information 2 in S1 File.

When both long covid and non-long covid groups were represented in a subtheme, they were split such that the data from the long covid group were reported first before the non-long covid group, as the non-long covid group helped to understand the context. A summary of the themes for each group can be found in Table 3 and further example of quotes in supplementary information 3 in S1 File.

**Table 3:** Summary of themes.

Theme	Long covid group	Non-long covid group
Long covid symptoms	Participants experienced physical (e.g., fatigue) and cognitive (e.g., brain fog) symptoms. Fatigue and breathlessness were the main barriers to RTW.	N/A
Changes in relationships	Long covid is misunderstood at work although support is received, which may result in guilt. Communication from managers and feeling valued are key for RTW. Family is a source of emotional and physical support for long covid symptoms. However, they could also be a distraction when WFH (e.g., homeschooling)	WFH and social distancing during the pandemic made interaction with others and work difficult. Communication and support from managers and support groups with colleagues were valued. Family was an important source of support throughout the pandemic but also a barrier to work, particularly due to homeschooling, which strained relationships.
Legislation, policies, and procedures	Long covid recognition and support by healthcare professionals were valued, but participants lacked confidence in the treatments that they received. At work, occupational health was supportive of long covid sufferers, and participants were appreciative of long sick pay. However, policies and procedures related to return to work were perceived as unsuitable for long covid. WFH and flexibility in hours worked and tasks were perceived as key to a successful RTW. Whilst measures to prevent the COVID-19 spread alleviated concerns of recurrent COVID-19-infection, participants felt more likely to get COVID-19 from mixing outside of work (e.g., supermarket)	Government restrictions during the pandemic affected work through increased perceived safety against COVID-19. Employer’s response to the pandemic and WFH was valued by participants, namely support in setting up home office.
Return to work	RTW translated in reasonable adjustments. Participants expressed the need to be retrained in their roles and sometimes considered leaving their profession.The slow RTW caused financial concerns. Overall anxiety regarding their job and perceived capabilities caused anxiety as participants reported difficulties accepting their new situation. Whilst RTW was perceived as a positive therapeutic step due to going back into a routine, RTW sometimes took place too early leading to further sickness leaves.	From WFH during/since the pandemic, work evolved through developing new ways of working. Depending on the industry, redundancy concerns and related financial difficulties caused anxiety, particularly for those who were furloughed, with financial implications. The fear of COVID-19 infection in relation to RTW after WFH caused anxiety, particularly from feeling more vulnerable to the virus as they had not been infected.

### Long covid symptoms

The theme focused on how the main symptoms experienced by those with long covid, namely physical (e.g., fatigue) and cognitive (e.g., brain fog) symptoms affected participants’ RTW. This theme was specific to the long covid group.

### Physical symptoms

Fatigue and breathlessness seemed to be the main barriers for returning to work with long covid. These symptoms affected getting ready for work, the commute, and physical activities at work in general:

*“I haven’t felt fit enough yet, I still get tachycardia when I’m moving around, I still, get short of breath on exertion.” (participant 2)*

Symptom management required flexibility around rest and physical activities (e.g., fewer physical activities following more intense days was an adaptation deemed beneficial).

*“It was helpful if I knew there was something intense happening that day, that I could follow a day not doing as much.” (participant 5)*

### Cognitive

Those with long covid experienced problems with memory, focussing, and "brain fog". Such cognitive impairments affected work as participants forgot what they are saying/doing or struggled with tasks requiring concentration (e.g., reading, writing):

*“The brain fog and the concentration […] you could get sent a four-page document to read and check and I struggle to get through one paragraph, I would have to re-read, re-read, re-read.”(participant 13)*

Concentration problems also affected people in the work setting, such that they needed to avoid potential distractions, mostly working around people. Participants preferred working in a quiet environment away from colleagues:

*“I can only concentrate for very short periods of time and that changes depending on how tired I am, and if somebody, […] I definitely couldn’t cope being in a room of people coming and going or the phone ringing and people talking to me and the computer on.” (participant 4)*

Cognitive impairment also reduced confidence in decision making and interactions with others, such that the memory issues made it hard to follow conversations and process information:

*“What if the brain fog and the memory loss is that bad that I make poor decisions or I say or do the wrong thing.” (participant 5)*

### Changes in relationships

Participants reported that long covid affected their relationships whether this was at work or at home, dependant on the level of understanding and support about their condition displayed from others. The non-long covid group also experienced changes in relationships during the pandemic, mostly resulting from social distancing, working from home, and home-schooling.

### At work

*Long covid group*. Long covid affected relationships, particularly at work. Most participants felt misunderstood by colleagues who may expect them to return to pre-covid work immediately, causing concerns about RTW:

*“I’ve felt there’s been some passive aggressive kind of comments and some kind of veiled saccharine sweet comments that haven’t been totally supportive.” (participant 2)*

Some participants experienced support from their team despite lacking an understanding of long covid, such support which sometimes resulting in feelings of guilt, and motivating premature RTW:

*“I felt guilty really, […] I was off for six weeks and I felt that I had improved to be able to return to some form of work.” (participant 11)*

Managers facilitated RTW, through regular communication, understanding the participant’s situation and symptoms, and being flexible. Manager support was translated into feeling valued and was salient to returning to work:

*“My managers have been really supportive, […]looking into research on long covid and things just to see how they can support me in getting back to work properly.” (participant 20)*

Contrastingly, lack of communication with managers made it harder for participants with long covid to express their difficulties. It was a clear barrier for the RTW as managers did not always understand the effect of the symptoms on participants’ ability to carry out their work, which needed to be explained:

*“They [managers] have said that if I’ve got any pressing issues then I can contact them, but they make you feel like your issues aren’t worth their time, so it’s a very, a very unsatisfactory situation.”(participant 8)*

*Non-long covid group*. The non-long covid group reported that the pandemic, particularly working from home and social distancing, affected interactions with others, making it harder to connect and relate to others.

*“It’s made it harder to sort of connect with people and I think colleagues that you work with because […] people are working from home and […] self-isolating and it kind of, affects the team.” (participant 27)*

Through having fewer interactions with others, they reported feeling less in control of their work as they were having to improve interpersonal skills and adapting to online relations (such as teachers not being able to support students face-to-face). This loss of control was most noticeable for teachers and the interactions with pupils.

*“You had the feeling that it was all out of your control as well because you couldn’t control anything the kids were doing or not doing, they could turn on a Teams lesson, I couldn’t see them or hear them, they could turn on a Teams lesson and literally not contribute.”(participant 33)*

Participants created communication groups and organised support meetings with colleagues to stay in contact as if they were seeing each other in the office, whilst offering a level of support, mainly for those who lived alone.

*“Just checking in with each other, we had a WhatsApp group but also we did little small group check-ins as well just to support each other when we could.” (participant 30)*

The effect of the pandemic on relationships with managers was largely influenced by recognition, guidance about the requirements for working from home and reassurance to ensure that their position was safe.

*“[Our relationship with the manager was] affected probably only in kind of positive ways really because it just made me see how the service and the management respond in a time of crisis, […] I can’t fault them they have been extremely supportive”. (participant 26)*

### Family

*Long covid group*. Families supported those with long covid via emotional support (having someone there) and practical support (taking over more responsibilities around the house and children) which facilitated the RTW. The value from family support was linked to witnessing and understanding the experienced difficulties:

*“They’re [who?] encouraging and also they know exactly what happened going through, they know exactly where I am at each point, so I think they’ve got the real picture, whereas people at work maybe have only got little snapshots and aren’t seeing me.” (participant 2)*

However, family was mentioned in terms of "demand" also, which affected work. With working from home and particularly with home-schooling, family acted as a distraction from work and caused tensions, thereby affecting family relationships. With that said, after becoming ill with COVID-19 and the associated fear of health complications, family became a priority over work.

*“We have all got conflicting demands at home, they seem to take precedence, clamouring children, very attention-demanding, so it’s been really hard to motivate myself to do the same level of attention to my work as I am used to doing in the office.” (participant 8)*

*Non-long covid group*. During the pandemic, the non-long covid participants reported caring for their vulnerable family members, making working from home a necessity.

Overall, family was seen as a form of support throughout the pandemic.

*“I guess support from the family, […] just perhaps contacting each other a bit more, that was something that my family did do, kind of distanced support if you like, that side of things, that side of support was a bit limited because I don’t have family here so it’s more difficult really” (participant 30)*

However, during the lockdown, because of home-schooling, family was an obstacle to working and affected relationships between family members.

*“It was ruining the relationship between me and my son really because I just turned into this really stressful nagging mum "oh have you done work? rah.” (participant 28)*

### Legislation, policies, and procedures

The experience of working and returning to work was associated with legislation, policies, and procedures. These may have been external to their place of work such as pandemic-related restrictions or the use of the healthcare system for the long covid group; or at work. For the non-long covid group, employers’ support focused on enabling them to work from home (e.g., with IT support) whilst for the long covid group policies and procedures were key factors to organise a suitable return.

### Outside of work

The long covid group acknowledged the support from healthcare professionals and health services at work as they aimed to coordinate the RTW or help with their mental health.

*“I’ve got some really nice information from my occupational health […] and it’s really sensible.” (participant 15)*

However, some healthcare professionals questioned their long covid condition and participants lacked confidence in the treatment received.

*“[At work, long covid] was accepted; but down at my local surgery, [healthcare professional] said to me, "how do you know you’ve got long covid"?”(participant 9)*

Participants reported that government restrictions related to the pandemic and associated adaptation from their employer affected their work and concerns around getting COVID-19, primarily working from home, wearing personal protective equipment (PPE) and vaccines:

*“I’ve had both of my injections [vaccinations], still wearing masks in lots of places, yeah, [getting COVID-19 is] not a significant worry for me personally.” (participant 16)*

### At work

*Long covid group*. This subtheme focused on the reaction of the employer to the participants’ sickness. Participants were grateful for long paid sickness leave, reducing financial constraints for returning to work before they are ready:

Varied support was received from employers, dependent on whether it was from occupational health or human resources. The former appeared supportive whilst the latter lacked an understanding of long covid, and displayed substandard communication within the department causing frustration to the participants:

*“The major obstacle was [human resources], the occupational health people were wonderful. I mean they were so on the ball.” (participant 9)*

Long covid participants did not find work policies and procedures in place suitable to their situation and their long covid symptoms. Participants were offered standard phased returns, which did not take into account the wide array of symptoms and their variations from day-to-day. In addition, given the long-lasting symptoms, they felt they needed longer to RTW than the standard four-weeks phased return they were commonly offered. This resulted in participants staying on leave for longer or having concerns around returning to sick leave due to their symptoms worsening from overworking:

*“I was working at home, it was a six-week phased return, and I was working from home and from 50% up to about 90% of my timetable, but then I had to go into school, so one day in school knocked me right back in terms of fatigue.” (participant 9)*

Participants reflected on what they felt worked or could have worked better to support their RTW, predominantly flexibility around the hours of work and work content. In addition, working from home was seen as a helpful form of flexibility. Such flexibility was perceived as another way for the employer to express support:

*“It’s got to be a flexible approach to how they manage to work and in what way they work and time.” (participant 5)*

Participants with long covid expressed that existing preventive measures to reduce spreading of the virus and the number of cases in the place of work facilitated RTW by reducing concerns around COVID-19. However, in some environments, such as schools, measures in place were not perceived as effective. Furthermore, some felt more likely to get COVID-19 from mixing with others outside of work, for example, going to the supermarket or from their school-aged children. The fear of COVID-19, whilst a concern, was generally not perceived as a barrier to returning to work, although some participants remained fearful of it:

*“I’m happy to go to the supermarket, I’m happy to go out and see people, so I don’t think that […] it’s not worrying me.”(participant 15)*

*Non-long covid group*. The non-long covid group commented on the employer’s response to the pandemic and the support they offered, for the most part, IT support. Some employers also offered access to a helpline for support around the pandemic.

*“We have the support in terms of sort of, helplines and we’ve had supervisors and there was like a specific sort of COVID helpline, if that makes sense.” (participant 27)*

Communication, support and promoting staff wellbeing was valued whilst the opposite reduced work engagement during lockdown or upon return from being furloughed, thereby causing uncertainties and negative feelings.

*“There was no contact when you were off, e[my employer] didn’t make contact with you […] didn’t reassure you, inform you where they were going or what was happening. Yes, so that was a tough time […] because you just didn’t know what was happening”. (participant 34)*

Working from home sometimes meant having to set up office/working space at home. At time not having a dedicated office space made it hard to switch between work and leisure:

The non-long covid group had to get equipment and software for working from home during the pandemic. Not having the right equipment influenced health as some employers were going through the motions to provide staff with what was needed:

*“I basically got tennis elbow in that arm from overuse of the mouse. Then I swapped using the mouse, they got me a special mouse”. (participant 24)*

### Return to work

For the non-long covid group, this theme related to working from home during the pandemic, being furloughed, and returning to pre-pandemic place of work. Through the pandemic, both groups experienced a change in their role, whether it was due to their health (long covid) or working from home. Returning to work evoked emotions such as anxiety. However, the long covid group also reported financial concerns linked to the slow return to work.

### Change in role

*Long covid group*. Returning to work with long covid symptoms meant that alternative tasks were suggested to manage participants’ symptoms and adapt to their current situation. For example, the nurses were encouraged to conduct more administrative tasks that they could do from home and were deemed less physical.

*“I was really starting to struggle with managing [my mood] and being stuck in the house and really isolated, so [my team leader] suggested [I] did clerical work instead.” (participant 3)*

Following a long break, some participants expressed the need to regain knowledge and skills to cope with symptoms or lack in confidence. On occasions, participants expressed a need to change job role because of their symptoms, or because their employer did not respond to their long covid symptoms as they were expecting.

*“There is absolutely no way I could go back to being a principal teacher […] I’m going to be struggling even to be able to teach, let alone to be the leader.” (participant 10)*

*Non-long covid group*. Through the pandemic the non-long covid group reported a change in their role at work. They had to develop IT skills due to working from home and teaching online whilst using IT tools (e.g., videoconferencing) with which they were not familiar, or having an opportunity to learn new skills to change their position.

*“It’s obviously changed the things we do, it’s opened up a lot of possibilities, but the technology that we have had to learn.” (participant 31)*

The non-long covid group expressed concerns about losing their job because of the pandemic. Depending on the industry, some participants experienced such concerns (e.g., those in administration service) whilst other did not (e.g., those in healthcare, building, IT):

Some participants were furloughed. With an uncertain future and related anxiety, some considered seeking a new position:

*“I had already been starting to make [contact about job opportunities] in industry […], I was thinking very much of worry and stress and anxiety.” (participant 33)*

The furlough scheme also added pressure on those who were not furloughed as they were allocated some of their colleagues’ work. Similarly, being back at work after being furloughed meant that they needed to catch up with their work:

*“My workload had increased […] because if you take annual leave, you go back, you have a number of things to action […] your colleagues were then on furlough so you were picking up their work.” (participant 33)*

### Financial situation

The slow RTW, the reduced hours, and the adjustments to the role for the long covid group, had financial implications and often resulted in loss of earnings through unpaid leave or reduced hours:

*“If you do a phased return you [are meant to mix] your hours with your annual leave and I think that’s really unfair.” (participant 1)*

Although some participants (particularly working for public organisations) commented on still being paid their normal wage, which was an important factor in the phased return:

*“I am lucky that I work for [public organisation] so I’m getting full pay at the minute but […] then I would go on to half pay, which would be a real difficulty for us*.” *(participant 2)*

### Feelings related to return to work

*Long covid group*. Participants experienced negative emotions linked to their RTW. For the long covid group, they were often characterised with anxiety and lack of confidence about being ready or able to perform their work because of their long covid symptoms. They also experienced difficulties with acceptance of their situation and their limitations:

*“Just that my job’s got a lot of responsibility, and I wouldn’t feel safe to return to such responsibility as my condition is unpredictable.” (participant 19)*

However, the long covid group also expressed a perceived increased immunity from having had COVID-19, and from the COVID-19 vaccines, which reduces those concerns by increasing their feeling of being protected against the virus:

*“I feel like I’m more protected because I’d had[COVID-19], and I’ll have some antibodies, I’ve had my first vaccination, so I feel like my body is probably better protected.” (participant 12)*

Returning to work may be beneficial for the long covid group given that it means getting back into their pre-COVID-19 routine. With that said, such motivation to RTW may delay recovery by returning to work too early. In addition, being off work for a long time triggered feelings of guilt, which further motivated their early RTW:

*“It was about trying to get some normality back and […] live a bit more of a normal life.” (participant 5)*

*Non-long covid group*. The non-long covid group experienced concerns about returning to work, sometimes linked to the uncertainties about the virus, after the first lockdown and working with people face-to-face in general. The concerns and the measures in place affected the way that people work. However, the later interviews (December 2021) which took place when many pandemic-related restrictions in the UK were lifted (e.g., social distancing, wearing a face-mask in public places) and the vaccines for COVID-19 were more widely available, showed a reduction in concerns with less comments about unknown and uncertainties. The lack of concerns seemed heightened by the fact that they had not caught COVID-19 so far:

*“We didn’t know very much at the beginning, so I was quite worried about getting it, and [COVID-19] was before I knew about long covid.”(participant 27)*

## Discussion

This study sought to explore the experiences of people’s RTW who had long covid and those who did not. People with long covid experienced difficulties to RTW due to long-lasting symptoms. Such symptoms affecting work activities predominantly revolved around fatigue, breathlessness, and cognitive problems affecting stamina, memory, and concentration. The symptoms experienced, coupled with difficulties returning to work, had an impact on their relationships at work, where recognition of their condition and support from colleagues and managers, often through regular communication, was critical to ensure a suitable return.

The pandemic affected work and relationships whilst also causing a variety of concerns around financial implications and job security for everyone but proved further challenging for those with long covid. At the time of interviews, long covid was not a recognised condition which further complicated relationships at work (e.g., feeling misunderstood) and participants lacked a suitable RTW plan. For the non-long covid group, difficulties at work during the pandemic were strongly related to WFH leading to changed workplace relationships due to not being with colleagues and managers regularly, and new workplace practices (e.g., working remotely and using videoconferencing). For the long covid group, work change was a necessity due to their symptoms in addition to the restrictions related to the pandemic. The non-long covid group interviews particularly contrasted with the long covid ones regarding fear of COVID-19 infection when returning to work, which was a significant concern for the non-long covid group, consistent with existing literature demonstrating such fear to be common during the pandemic [37,38].

Post-exertional malaise, marked by a worsening of symptoms after physical or mental exertion, has been identified as a significant aspect of long covid [39,40]. Research indicates that physical activity can exacerbate long covid symptoms and is identified as a substantial obstacle for the RTW [41]. These findings align with our participants’ experiences, who shared that returning to work prematurely could lead to a relapse, consequently further delaying their RTW. Therefore, they emphasised the crucial role of employer flexibility in supporting them to manage physical efforts and symptoms, which could not only support RTW but also prevent job loss resulting from long covid [42].Long covid symptoms caused cognitive overload and difficulties in learning new skills in addition to physical difficulties that could not always be accommodated due to the nature of the work (e.g., healthcare keyworkers). However, through support from their employers and reasonable adjustments, there was no report that their job was at risk. In contrast, except for those working for public organisations and education, the non-long covid group reported concerns regarding work uncertainty, particularly with the furlough scheme. The findings on anxiety related to job-loss risk during the pandemic are consistent with existing literature linking perception of job insecurity to anxiety [43,44], particularly during the pandemic given its impact on the economy resulting in the rise in unemployment rates [45].

The study confirms the importance of returning to work after recovery from acute illness to help individuals get back into their pre-COVID-19 routine and increase quality of life whilst gaining a sense of recovery [16]. Those in physically demanding roles are more likely to take on a different position due to ongoing symptoms [16]. In this study participants’ work often changed to include more administrative or desk-based tasks and they sometimes called for assistance for the more physical aspects of their role.

Fatigue, lack of concentration and breathlessness were the most recurrent symptoms delaying the return to work, thereby confirming previous findings [32]. Returning to work after long covid differs from most post-critical illness syndromes because the symptoms vary from one person to another and from day-to-day, and relapse, particularly resulting from premature RTW. Consequently, standard pre-defined phased returns are often not suitable for long covid as participants reported the need for a flexible return plan with regular reviews. Participants also reported that the phased return should be spread over a longer period than the commonly-offered four-weeks. Without suitable vocational rehabilitation, long covid may contribute to the rise of people falling out of work or on long sickness leave [19] thereby highlighting the need for specialist help to support those with long covid to RTW.

Whilst breathlessness minimised participant’s ability to carry out physical activities, in addition, fatigue varied periodically and increased the difficulty to follow scheduled work. Evidence shows that fatigue affects daily functioning and performance at work as the feeling of exhaustion may impair physical and/or cognitive functioning [46]. Here, cognitive difficulties took form of brain fog and worsened short term memory thereby reducing participant’s confidence at work (e.g., to make decisions). Such difficulties are further problematic in healthcare professions where there is an element of risk for both staff and patients (e.g., manual handling), as reported in a systematic review of the effect of nurse fatigue. As such, in our sample, support in form of assisting staff was found beneficial to reduce physical activity and provide shift cover on days participants were not able to work. In addition, regular breaks helped fatigue management, and working in an isolated area to avoid interruptions from others, or WFH were useful to increase concentration and focus. Overall, given that participants’ experiences varied depending on their symptoms and work situations, human resources and managers’ understanding of their condition is crucial to provide adequate support for a successful return to work and to avoid relapse.

Premature RTW was reported as a common cause of relapse. Previous findings demonstrate that pressure from colleagues who did not understand their condition motivated the early return [22]. In our study, in addition to pressure from colleagues, participants reported feeling guilty, pressurising themselves from a sense of failing their colleagues or concern about how others perceived their absence. Guilt and presenteeism are particularly prevalent amongst healthcare workers [47,48], which may have been intensified by the pandemic, given that many of our participants were frontline workers and in demand at the time of interviewing, which is in line with previous research [49]. Given that sickness presenteeism can also have significant effects on mental health and is more prominent since the COVID-19 pandemic [50], motivation for returning to work is an important consideration and should be explored to avoid an unnecessary premature return which may further delay recovery.

Whilst some participants in the long covid group expressed concerns about the fear of reinfection, the majority indicated that it did not impede their RTW process. This was attributed to the belief that the risk of reinfection primarily emanated from exposure to the virus outside of the workplace, and their confidence in the preventive COVID-19 measures in effect during the interviews (e.g., social distancing, face masks). However, given that there are presently no preventive measures in place, despite a decreased risk of developing long covid after recurrent infection [51], those with long covid may have increased fear regarding RTW, which has since been demonstrated [52].

### Strength and limitations

Whilst aiming to explore the experience of returning to work for those with long covid, the non-long covid group who never had COVID-19 were interviewed. As the pandemic affected the general public’s work situation [53], some difficulties might have been due to public health restrictions to restrict COVID-19 infection rather than specifically due to long covid. As such, the interviews of the non-long covid group describe work challenges during the pandemic thereby providing context to differentiate the effects of the pandemic and of long covid in relation to return to work with long covid.

All our long covid participants had tested positive for COVID-19 and experienced residual symptoms after recovery. However, we did not enquire about hospitalisation or their experience of care during the COVID-19 infection itself. Evidence suggests that hospitalisation and experience of critical care are important factors when considering quality of life and return to work following critical illness since return to work can be longer and people may experience additional symptoms related to trauma [54].

We explored the effects of symptoms on return to work, not just in terms of their nature but also their time course and fluctuation in severity. However, irrespective of the severity of the symptoms, if people with long covid felt that they couldn’t work, there may have been other reasons contributing to their inability to return to work such as longer standing relationships with the employer or loss of opportunity as a result of the pandemic or becoming ill.

Our sample mainly represented women working in healthcare and education, in the public sector with little representation of other job categories. Whilst long covid has had a significant impact on numerous women employed in the healthcare and education sectors, [8], other employees may report different experiences of returning to work with long covid.

In their study on critical illness, Rogers et al. [32] described common symptoms of trauma as barriers to RTW. These were not spontaneously or explicitly reported in our sample. However, our topic guide did not refer to hospitalisation and our sample was recruited form the general population rather than those experiencing critical care or hospitalisation.

This study was performed in the United Kingdom which has its own legislation and organisational structures in relation to work and healthcare as well as public health pandemic restrictions. These are likely to bear some resemblance to the same factors in other similar countries but may be much less relevant to other countries, especially lower and middle income.

We acknowledge that our recruitment strategy may have attracted a particular group of people to self-select to participate in our study. This, therefore, would have influenced our findings, and potentially reduces the ’transferability’ of our study to other groups. However, this issue is not unique to our study, but is an issue that is inherent to most interview studies [55]. Furthermore, recruiting through social media may have inadvertently excluded potential participants who lacked access to the internet, resulting in a sampling bias towards individuals who are more likely to be higher income and internet access [56]. Consequently, participants recruited through this method may have had different experiences with RTW, particularly in relation to long-term disability benefits. Higher-income individuals may have been more likely to have access to paid long-term disability benefits, which could have influenced their RTW experiences compared to lower-wage hourly workers who may not have had access to such benefits. They may also have more control over their working conditions as higher paid posts are often associated with greater autonomy in the work place. This potential bias limits the generalisability of the study findings.

### Clinical implications

As long covid is now a recognised condition (but was not recognised at the time these interviews were conducted), employers are required by law to consider reasonable adjustments after the assessment of the person, their symptoms, and the demand of their work so that they can RTW or remain at work [57]. Given the main barriers to RTW highlighted here are fatigue, cognitive difficulties and breathlessness, those with long covid in professions involving physical activities or long periods of concentration may require such adjustments. Employers may acknowledge difficulties through integrating less physically demanding tasks, providing more time to complete tasks, allowing more breaks, or offering access to some support (assisting staff). Besides, given that symptoms may vary over the course of a week, employer flexibility is crucial to ensuring a sustainable RTW. A degree of home working may alleviate concerns related to commuting to work and allowing staff to take regular breaks may enable fatigue management, although some roles especially involving physical activities do not lend themselves to home working. Moreover, opportunity for home working could facilitate working in a quiet environment which was deemed important to help with difficulty concentrating.

Relationships both at work and at home need to be considered in RTW plans for those with long covid. Work colleagues, line managers, and human resources staff may need to be educated about long covid and its symptoms so that they are able to understand and better support those returning to work. Participants commented on the importance of family support but also that on some occasions, family commitments may contribute to worsening the symptoms thereby further emphasising the importance of family support. As such, interventions should consider patients’ situations holistically to review their support network as well as commitments outside of work.

## Conclusion

The study suggests that, beyond the pandemic and associated restrictions, long covid symptoms may affect RTW and recovery. The variability of the symptoms and the risk of relapse separate long covid from post-critical illness syndrome. Such findings suggest that there is a need for the development of a specialist vocational rehabilitation intervention tailored to long covid symptoms and roles available to the employee and employer, based on our suggestions. Such an intervention should include education to the patients, employers, and colleagues about long covid, in addition to support to manage symptoms. Given the importance of flexibility from the employer, communication from managers and human resources is of great importance to design and facilitate a tailored return plan for those with long covid and ensure a successful RTW.

## Supporting information

S1 File (DOCX)
