# Supplementary material for: Returning to work with long covid in the UK during lockdown and other COVID-19 restrictions: A qualitative study
Source: PLoS One. 2024 Aug 12;19(8):e0307062. doi: 10.1371/journal.pone.0307062 (PMC11318866; doi:10.1371/journal.pone.0307062)
Supplement: S1 File — (DOCX) [file pone.0307062.s001.docx]

Supplementary Information

# Supporting information 1 – Topic guide

A -For long covid

| **Questions** | **Prompts** |
| --- | --- |
| What is your work?  Since you became ill, have you attempted to return to work?  *(If returned to work)*  Reflecting on your (attempted) return, what do you think has worked well and what did not help? | -How long after recovering was it?  -Factors which motivated the decision to return then?  - Any support received (employer, family, colleagues)  -Adaptations/equipment  -Job accommodations (changes in roles, shifts pattens, breaks  -home working  -Flexibility around hours  -Work place environment changes  -reduce risk of infection  -changes in policies  -lone worker policies  -safeguarding policies  -communication methods |
| What do you think is a challenge to you returning to work?    How would you describe the feelings/symptoms *(use participants’ own words*) that you experience and how often do you experience them? | -Pressure linked to work performance  -Physical struggle  -Relationship with line manager  -Fear of getting ill (COVID-19) again  -Claim  -Feeling different, still ill  -Ability to return and do the same work as prior illness  -Changes in motivation  -Continuous or episodic  -Commute |
| What do you think would help to get you back to work? | -Employer support  -Gradual return  Political support- government drivers / policies  Financial support (eg government aid/ grants)  -Educating colleagues  -Regular physical assessment with return to work and tasks based on results |

B- For the control group

| **Questions** | **Prompts** |
| --- | --- |
| What is your occupation or what was it on the 01/03/2020?  How has the COVID-19 pandemic affected your work? | - Furlough scheme  - Any support received (employer, family, colleagues)  -Adaptations/equipment  -Job accommodations (changes in roles, shifts pattens, breaks  -home working  -Flexibility around hours  -Work place environment changes  -Reduce risk of infection  -Changes in policies  -Lone worker policies  -Safeguarding policies  -Communication methods |
| What do you think is a challenge for you to work in the current climate?    (If furloughed: What do you think represent a challenge for you to go back to work) | -Pressure linked to work performance (e.g., do more work with less staff)  -Fear of redundancy  -Relationship with line manager  -Fear of getting ill (COVID-19)  -Changes in motivation  -Going back to work after being furloughed?  -Struggle to find employment  -Industry of work |
| (If furloughed: what do you think would help to get you back to work?) | -Employer support  -Gradual return  -Political support- government drivers / policies |

# Supporting information 2 – Themes and subthemes cross-referenced to the ICF core set

| **Themes** | **Sub-themes** | **ICF** | **Quote** |
| --- | --- | --- | --- |
| Long covid symptoms | Physical | Energy and drive functions. | “Because of how severe the fatigue is and the amount of rest that I need, it would really impact me being able to do both the mental and physical aspects of my role.” |
|  |  | Exercise tolerance functions. | “It was helpful if I knew there was something intense happening that day, that I could follow a day not do as much.” |
|  | Cognitive | Higher-level cognitive functions | “I wouldn't feel safe enough to make the decisions and communicate with people with how my brain functions at the minute.” |
| Changes in relationships | At work | Complex interpersonal interactions. | “I've felt there's been some passive aggressive kind of comments and some kind of veiled saccharine sweet comments that haven't been totally supportive.” |
|  |  | People in position of authority | “They are letting me explain how my symptoms are affecting me at home and how they are affecting me at work and allowing me to kind of decide how I want to return.” |
|  | Family | Immediate family | “At the moment it's really my husband and the children doing the housework and things because I'm slowly starting to do more again because I want to see if I can do housework.” |
| Legislation, policies and procedures | Outside of work | Healthcare services, system and policies | “It probably needs to be seen as a recognised condition by employers and by the government.” |
|  | Work | Labour & employment services system and policies | “[Human resources] didn't seem to pass on any notes to each other so you were starting afresh every time.” |
| Return to work | Change in role | Acquiring in skills | “I was really starting to struggle with managing [my mood] and being stuck in the house and really isolated so she suggested [I] did clerical work instead.” |
|  |  | Acquiring, keeping and terminating job | “There is absolutely no way I could go back to being a principle teacher […] I'm going to be struggling even to be able to teach, let alone to be the leader.” |
|  | Financial situation | Economical sufficiency | “If you do a phased return you [ are meant to mix] your hours with your annual leave and I think that's really unfair.” |
|  | Stress and feelings | Handling stress and other psychological demands. | “I feel like I'm more protected because I'd had it, and I'll have some antibodies, I've had my first vaccination so I feel like my body is probably better protected.” |
|  |  | Emotional functions. | “Just that my job's got a lot of responsibility and I wouldn't feel safe to return to such responsibility as my condition is unpredictable.” |

Supporting information 3 – additional quotes to illustrate themes and sub-themes

| **Themes** | **Sub-themes** | **Long covid group** | **Non-long covid group** |
| --- | --- | --- | --- |
| Long covid symptoms | Physical symptoms | “Because of how severe the fatigue is and the amount of rest that I need, it would really impact me being able to do both the mental and physical aspects of my role.” (participant 1)  “[When] teaching, you have to do quite a lot of voice projection and I'm finding that I get breathless quite a lot.” (participant 10)  “I'm even a bit anxious about the travelling to work because it's about a 25-minute drive, when I get there walking from the car park to my office, having to get changed [...] that's going to be a bit of tiring out before I start.” (participant 2)  “I don't do any direct care support [anymore], […] I'm never out in services, I historically would push wheelchairs, [handle patients], don't do any of that now.” (participant 5)  “[I] need to step out for a bit or take a break and that needs to somehow be factored in because if the fatigue hits, it hits really hard, if I do too much, the following day the impact is quite a lot; so, I've got to measure out my limits really.” (participant 5) |  |
|  | Cognitive | “I have lots of lists everywhere, I'm very much a list-maker these days. As soon as the idea comes into my head, I write it down somewhere.”(participant 9)  “Sometimes I may well not quite grasp what [my colleagues] are saying or have to spend a bit longer trying to work out what they're saying.” (participant 21) |  |
| Changes in relationships | At work | “[Colleague] had COVID the same time as me but had been absolutely fine after it, so I felt she wasn't very empathic.”(participant 1)  “People […] kind of forget you've been off so I'm a bit worried about that, not being expected to be on full power straight away.”(participant 2)  “I don't think all of my colleagues fully understood before I went off […] but I do feel that I will be supported when I do go back.” (participant 1)  “I think just regular contacts and seeing how he [manager] could help me and to be fair, he would have just let me work whatever hours, wherever I needed to at whatever time.” (participant 13)  “I've spoken to my [manager] in a way regarding making mistakes with brain fog, I said yes I'm happy to go on surgery but if I do a mistake, it could cost an animal's life.”(participant 7) | “A lot of young people have become disengaged with the service.”(participant 25)  “[Communication with colleagues] was more about checking in with each other and just making sure that people were okay, because some of my colleagues lived on their own, so I was pretty much checking in on those ones.” (participant 29)  “Part of the joy of [teaching in a classroom]is going around and helping them one-to-one and seeing those sparks, moments of when they really get something, and you can't do that when you are stood in a circle at the front of the room.” (participant 31)  “We carried on really keeping in touch with each other really regularly through that WhatsApp group and that was really helpful and sometimes if people were kind of "oh gosh has anyone dealt with this kind of presentation before" and people would reply and say "oh yes, I have had a client like that" and then it is like "oh lets set up a call because I'm not quite sure what I am doing." We might ring each other” (participant 28) |
|  | Family | “When I'm struggling with my symptoms [and I am] quite low, depressed, […] having [family member] around helped me get through some pretty tough moments over the last year and I am sure that's helped me continue with work when I've needed to.”(participant 6)  “At the moment it's really my husband and the children doing the housework and things because I'm slowly starting to do more again because I want to see if I can do housework.”(participant 7)  “I went back for six weeks on a phased return in a way […]but it was not on sick pay or anything, it was the holiday taken because it was due to children being at home so when I was at home I did home schooling the children” (participant 7)  “I was scared for a period of time that I wasn't going to live and not see my children grow up […]I would prioritise my own health more and my family's needs [now]” (participant 19) | “Working from home I was very reluctant, I didn't actually want to work from home, but I had to consider my mum's health” (participant 29)  “As my own children were at home at the time, it was quite mentally challenging trying to deliver lessons on-line whilst supporting them with their own schoolwork, they are quite young so it was quite difficult to juggle the two things at the same time. It just made me not want to do the job any more, if I am completely honest.”(participant 31)  “There were times when I was home schooling for some days as well as trying to work in the first lock down and that was pretty stressful.”(participant 28) |
| Legislations, policies and procedures | Outside of work | “I've also been put in touch with an organisation through my work […] which is a counselling organisation that provides six free sessions through my employer, so I'm taking advantage of that.” (participant 10)  I don't feel very confident in what [GPs] have done, they haven't really supported me […] so I kind of think, you know what it's up to me to make myself better and use all the skills that I have to try and manage my own condition but I just didn't bother [contacting my GP] any more. (participant 10)  “I suppose PPE was probably the main thing that changed, especially at the beginning and then the guidance came out because now we have to obviously wear masks in the office […] it still continues to change even now as rules change.” (participant 17)  “I felt we were safe enough away from students, you know the desks were far enough away and we even had like boxes that we had to stand in or lines that we couldn't go past to meet the students, so no I felt safe.” (participant 22) |  |
|  | At work | “We have […] employment schemes to support us financially […] so I think if you are a low paid worker maybe that might not be the case and people might not be supported but I certainly do feel in [public organisation] that we have been.” (participant 11)  “Occupational health have been very supportive and said that even though they want me back at work, they want me back at work to stay back at work so they want me back at work at the right time.” (participant 2)  “[Human resources] didn't seem to pass on any notes to each other so you were starting afresh every time.” (participant 2)  “If I felt that I couldn't sustain that phased return […] they could have given me work, you know, like a desk job at home, like monitoring COVID positive patients with a laptop, but I didn't really want to do that.” (participant 11)  “I think in professions where you could be allowed to work from home that would be ideal for people with long covid.” (participant 9)  “They were very supportive of me having days off when I needed them.” (participant 6)  “I'm not worried because we work, I was one positive case and no-one else got it because we have quite a [a lot] of PPE.”(participant 7)  “The school adopted many COVID procedures but it's a big place with lots of people and lots of children who […] avoid the rules outside the school so whilst they were obeying rules in school, what they were doing out of school made it a bit of a nonsense: schools just aren't safe places.” (participant 9)  “My kids are back at school, they've got a chance of bringing it home, that doesn't bother me. I don't think the worry of getting [COVID-19] again is a hindrance of me getting back to work.” (participant 15) | “We had to move to working online; so I had been seeing people only face to face up until that point and then we had to switch.” (participant 28)  “I was working in my living room at home before, which psychologically, that was kind of making things worse as well, so it kind of prompted me when I got back home to convert the conservatory where I am living into an office so I had a different space to go into.” (participant 26)  “They asked me if I needed any equipment, and I requested a wrist mouse mat thing.” (participant 24)  “I was working at like a kitchen table at home initially and that wasn't great because it wasn't the right height and they eventually got me a desk.” (participant 28) |
| Return to work | Change in role | “I'd probably have to re-learn parts of my job because when I think back to what did, I do on a day-to-day basis, I feel like I can't remember.” (participant 19)  “[Following employer’s lack of support about long covid] I have even looked at perhaps a career change […] Not because I do not love the job that I do, but because I feel undervalued.”(participant 17) | “I started doing my own training, because you were allowed to do training during furlough, so I started to have a purpose.” (participant 34)  “We are still going through a re-structure and that's happened over the past six to eight months, I would say and initially I was worried that it was going to affect my team.” (participant 25)  “I think working in mental health and understanding what the pandemic has done to everybody, I think I've probably got a secure job for the rest of my life!” (participant 24)  “In fact, increase in demand in some industries was a challenge given the volume of work.” (participant 34)  “I think for me really the only challenge is just sort of ongoing work which in the building trade seems to have been an abundance of work.” (participant 34)  “People's mental health which in turn affects the way they work and anxiety and a sense of uncertainty about the future and things like that can really affect the way that somebody can work.” (participant 27) |
|  | Financial situation | “The sick pay that I was getting by that point was really poor.”(participant 5) |  |
|  | Feelings related to return to work | “The difficulty is being accepting that this is your limitation, and it doesn't matter what you want, this is where you are […], it's so boring and frustrating.” (participant 15)  “I'm too motivated to return to work, that it's been detrimental. I think I've tried too hard to stay in work which is why it has made everything worse […] I was just dragging myself to work even though I felt terrible because I didn't know what else to do.” (participant 18)  “I guess sometimes I feel guilty, but I just feel that I needed that time to get there, it's been a long process, I haven't been particularly ill.” (participant 11) | “[Fear of COVID-19] was quite a large factor when I did need to do any work with colleagues in person, […] it was the whole people learning how to behave in that situation, managing the public transport systems in that situation and the precautions, the PPE and everything like that was so new.” (participant 8)  “I don't seem to catch colds or I'm not generally ill from most things usually, so I wasn't overly concerned about catching it to be honest.” (participant 29) |
